# Effective multi-modal clustering method via skip aggregation network for parallel scRNA-seq and scATAC-seq data

**DOI:** 10.1093/bib/bbae102

**Published:** 2024-03-16

**Authors:** Dayu Hu, Ke Liang, Zhibin Dong, Jun Wang, Yawei Zhao, Kunlun He

**Affiliations:** School of Computer, National University of Defense Technology, No. 109 Deya Road, 410073 Changsha, Hunan, China; School of Computer, National University of Defense Technology, No. 109 Deya Road, 410073 Changsha, Hunan, China; School of Computer, National University of Defense Technology, No. 109 Deya Road, 410073 Changsha, Hunan, China; School of Computer, National University of Defense Technology, No. 109 Deya Road, 410073 Changsha, Hunan, China; Medical Big Data Research Center, Chinese PLA General Hospital, No. 28 Fuxing Road, 100853 Beijing, China; Medical Big Data Research Center, Chinese PLA General Hospital, No. 28 Fuxing Road, 100853 Beijing, China

**Keywords:** single-cell clustering, skip aggregation network, denoising autoencoder, ZINB, deep learning

## Abstract

In recent years, there has been a growing trend in the realm of parallel clustering analysis for single-cell RNA-seq (scRNA) and single-cell Assay of Transposase Accessible Chromatin (scATAC) data. However, prevailing methods often treat these two data modalities as equals, neglecting the fact that the scRNA mode holds significantly richer information compared to the scATAC. This disregard hinders the model benefits from the insights derived from multiple modalities, compromising the overall clustering performance. To this end, we propose an effective multi-modal clustering model scEMC for parallel scRNA and Assay of Transposase Accessible Chromatin data. Concretely, we have devised a skip aggregation network to simultaneously learn global structural information among cells and integrate data from diverse modalities. To safeguard the quality of integrated cell representation against the influence stemming from sparse scATAC data, we connect the scRNA data with the aggregated representation via skip connection. Moreover, to effectively fit the real distribution of cells, we introduced a Zero Inflated Negative Binomial-based denoising autoencoder that accommodates corrupted data containing synthetic noise, concurrently integrating a joint optimization module that employs multiple losses. Extensive experiments serve to underscore the effectiveness of our model. This work contributes significantly to the ongoing exploration of cell subpopulations and tumor microenvironments, and the code of our work will be public at https://github.com/DayuHuu/scEMC.

## INTRODUCTION

The advancements in single-cell transcriptomic sequencing technology have revolutionized transcriptome analysis, enabling biologists to delve into cellular heterogeneity with remarkable resolution at the single-cell level [1–3]. Clustering analysis plays a pivotal role in transcriptome analysis, allowing for the unsupervised identification of cell subpopulations, which is crucial for downstream analyses.

Over the years, numerous attempts have been made to develop clustering methods for single-cell data [4, 5]. Initially, the focal points of research revolved around fundamental clustering models such as $k$-means clustering and spectral clustering [6, 7], along with their enhanced variants. For instance, Chen *et al*. proposed a weighted soft $k$-means clustering model tailored for single-cell data, replacing the original hard clustering with a soft one [8]. While these methods achieved some success, they often struggled to extract nonlinear features from the cell interactions. With the development of deep learning, researchers began to explore the realm of deep neural networks for clustering analysis. Notably, DESC emerged as a representative work, utilizing neural networks to learn meaningful representations while effectively mitigating batch effects [9]. Furthermore, scDeepCluster employed an autoencoder network to concurrently conduct noise reduction and clustering for single-cell data [10]. These deep clustering approaches made significant progress but overlooked the topological information among cells. In response to this limitation, graph-based deep clustering algorithms were developed, benefiting from the interactions among cells. Chen *et al*. introduced scGAC [11], which employed a graph attention network to execute clustering analysis. Gan *et al*., recognizing the significance of both attributes and topological information, proposed a deep structural clustering model scDSC [12], capable of simultaneously addressing these aspects. Despite their promising performance, these single-modal methods encountered limitations when handling multi-modal single-cell data.

Multi-modal single-cell data refers to the data obtained by sequencing the same batch of cells using different omics technologies [13–16]. Currently, the parallel analysis of single-cell RNA-seq (scRNA) and single-cell Assay of Transposase Accessible Chromatin (scATAC) is a common scenario. With the rapid development of sequencing technologies, the availability of multi-modal data is increasing. By leveraging the distinct modalities of the same cell, we can gain more comprehensive insights into cellular states. In recent years, several parallel clustering methods have been developed for scRNA and scATAC data. For instance, scMVAE presents a Multimodal Variational Autoencoder (MVAE), imbued with three learning strategies for inferring the distribution of multi-modal cell data [17]. This field has seen extensive use of MVAE, Gong *et al*. utilized datasets of various modalities as inputs, applying MVAE for joint representation estimation, and performing clustering and visualization on the derived representation [18]. Simultaneously, Cao *et al*. introduced the SAILERX deep learning framework, which diverges from conventional approaches [19]. This method promotes local structural similarity between the modalities through paired similarity assessments, thereby effectively diminishing the impact of noise signals. Furthermore, Xu *et al*. developed a transfer learning method to identify generalizable chromatin interactions in scATAC-seq data [20]. Moreover, DCCA introduces an ingenious cycle attention model, designed specifically for the unified analysis of multi-omic cell data [21]. Inspired by the principles of subspace clustering, scMCS extends it to the realm of single-cell clustering [22], enabling the effective clustering of parallel single-cell data by diligently minimizing redundancy across subspaces.

However, prevailing parallel clustering methods for scRNA and scATAC often overlook the fact that scATAC data exhibit lower information richness compared to scRNA data [23, 24]. They treat the data from both modalities as equal inputs, disregarding the inherent differences in data effectiveness and sparsity. Consequently, in many cases, the clustering performance of the fused data from both modalities is even inferior to using only the scRNA data. This could be attributed to the low information richness of the scATAC modality, where the fusion process is challenged by the sparse information in scATAC data, resulting in poor quality of the aggregated cell representations for clustering. Existing methods face challenges in effectively integrating parallel scRNA and scATAC data, concurrently struggling to adequately fit the real distribution of single-cell data.

In light of the aforementioned points, we develop an effective multi-modal clustering model (scEMC), which integrates parallel scRNA and scATAC data while ensuring the quality of the aggregated cell representations. The proposed skip aggregation network (SAN) network extracts structural information from multiple modalities and facilitates cross-modal information fusion, simultaneously connecting with scRNA modality data via skip connection to promise that the fused cell representations do not suffer significant performance degradation. Additionally, to accurately model the distribution of real single-cell data, we have devised a Zero-Inflated Negative Binomial (ZINB)-based denoising autoencoder accompanied by a joint optimization module. Experimental results on five benchmark datasets demonstrate the stability and superior performance of the scEMC method, outperforming eight other baseline methods. The contributions of our work can be summarized as follows:

- We propose an effective parallel clustering framework scEMC, which mitigates the impact of unbalanced information richness of scRNA and scATAC data.
- Different from previous methods, we have introduced a pioneering SAN module that incorporates transformer structure to learn the global structural relationships between diverse feature spaces, facilitating aggregation across different modalities. Moreover, we create a skip connection between the aggregated representation and the scRNA modality data to safeguard the network from degradation.
- By leveraging a denoising autoencoder based on the ZINB loss, scEMC enables the network to fit the real distribution of single-cell data. Extensive experiments demonstrate the excellence of scEMC, surpassing the other benchmark methods.

## MATERIALS AND METHODS

### Preliminary

Multi-modal single-cell data refer to data obtained from the sequencing of the same batch of cells using multiple sequencing technologies. In this study, our primary focus lies in the parallel clustering of scRNA and scATAC data. Parallel clustering involves the simultaneous preprocessing of scRNA and scATAC data, followed by feature integration. scRNA and scATAC are interrelated in the processing phase, rather than being completely independent. To facilitate the illustration, we provide a clear mathematical description for them. The scRNA data is represented as $\mathbf{X}^{r}=\{\mathbf{x}_{1}^{r};...;\mathbf{x}_{N}^{r}\}\in \mathbb{R}^{N \times D_{r}}$, while scATAC denoted as $\mathbf{X}^{a}=\{\mathbf{x}_{1}^{a};...;\mathbf{x}_{N}^{a}\}\in \mathbb{R}^{N \times D_{a}}$, where $N$ signifies the number of cells, $D_{r}$ and$D_{a}$ denote the feature dimensions of the scRNA and scATAC modal data, respectively.

### The framework of scEMC

**Figure 1:**
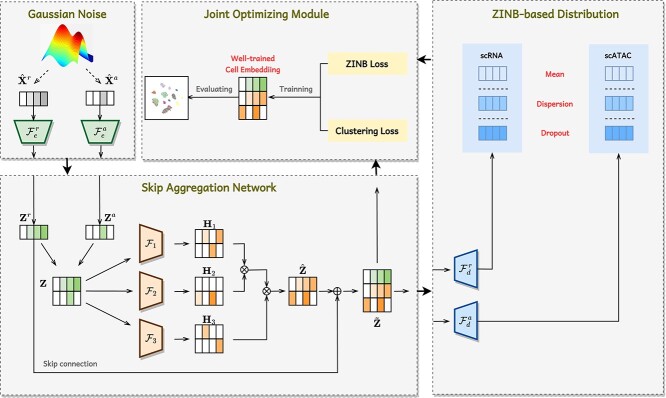
Illustration of the framework of scEMC. The whole process is divided into four stages. Following the introduction of Gaussian noise, the original numerical matrices of scRNA and scATAC data are input into an autoencoder. Afterward, the cell embeddings $\mathbf{Z}^{r}$ and $\mathbf{Z}^{a}$ from both modalities undergo effective fusion via an SAN, with a focus on preserving the utmost information from the informative scRNA modality. The final cell representations $\tilde{\textbf{Z}}$ derived from the SAN network are decoded to produce three data distributions. These distributions are employed to calculate the ZINB loss, which is jointly optimized alongside the clustering loss of the representations.

The architecture of scEMC aims to learn effective cell representations across multiple modalities and mitigate the impact of imbalanced data richness in diverse modalities, which is crucial for conducting parallel clustering. As illustrated in Figure 1, it consists of two main modules: a ZINB-based denoising autoencoder for generating cell representations and an SAN module for aggregating multi-modal information and preventing network degradation. For clearer understanding, the notations are presented in Table 1.

**Table 1:** Notation summary

**Notation**	**Explanation**
$\hat{\textbf{X}}^{r},\hat{\textbf{X}}^{a}$	Input data for for the scRNA and the scATAC.
$\mathcal{F}^{r}_{e},\mathcal{F}^{a}_{e}$	Encoders for the scRNA and the scATAC.
$\mathbf{Z}^{r},\mathbf{Z}^{a}$	Embedding for the scRNA and the scATAC.
$\textbf{Z}$	Embedding after concatenation.
$\mathcal{F}_{1},\mathcal{F}_{2},\mathcal{F}_{3}$	Encoders for computing structural relationship.
$\mathbf{H}_{1},\mathbf{H}_{2},\mathbf{H}_{3}$	Embeddings for computing structural relationship.
$\hat{\mathbf{Z}}$	Embedding after aggregation.
$\tilde{\mathbf{Z}}$	Embedding after skip connection with $\mathbf{Z}^{r}$.
$\mathcal{F}^{r}_{d}, \mathcal{F}^{a}_{d}$	Decoders for the scRNA and the scATAC.

The implementation process can be divided into four steps:

- First, the original scRNA and scATAC data, augmented with simulated noise, are fed into the denoising autoencoder. Subsequently, they are embedded into a lower-dimensional space, and the resulting embeddings are concatenated to build a shared embedding $\mathbf{Z}$.
- Inspired by the transformer architecture, $\mathbf{Z}$ is mapped into three independent feature spaces. We retain one of them ${\mathbf{H_{3}}}$ for learning transformations of the original features, while the other two ${\mathbf{H_{1}}}$ and ${\mathbf{H_{2}}}$ are used to compute global structural relationships among cells. This process results in the generation of a global structural enhanced embedding, denoted as $\hat{\mathbf{Z}}$. It is subsequently concatenated with the original scRNA embedding through skip connections, aiming to preserve the information-rich scRNA modality data. This yields the aggregated skip embedding $\tilde{\mathbf{Z}}$.
- The embedding produced by the SAN module then undergoes an intuitive decoding process, where it is decoded into distinct modalities using two separate decoders.

- Finally, three distributions, namely Dropout, Dispersion and Mean, are computed from the decoded embeddings. These distributions are then utilized to calculate the ZINB loss for different modalities. It serves as the reconstruction loss, which, together with the clustering loss, jointly optimizes the cell representations. By leveraging end-to-end training and real-time optimization, we obtain high-quality cell representations capable of achieving unsupervised clustering with high accuracy.

### ZINB-based distribution

To simulate the distribution of real cells and learn effective cell representations, we employ a denoising autoencoder based on the ZINB loss. Since real-world single-cell data often contain noise, we augment the input multi-modal data $\mathbf{X}^{r}$ and $\mathbf{X}^{a}$ with Gaussian noise. The process is denoted as follows: 


(1) \begin{align*}& \begin{aligned} \hat{\textbf{X}}^{r} =\mathbf{X}^{r} + \sigma_{r}*\mathbf{n}_{r};\hat{\textbf{X}}^{a} =\mathbf{X}^{a} + \sigma_{a}*\mathbf{n}_{a}, \end{aligned}\end{align*}

where $\mathbf{n}_{r}$ and $\mathbf{n}_{a}$ represent the simulated Gaussian signals added to the scRNA and scATAC data, respectively, with a mean of 0 and a variance of 1. $\sigma _{r}$ and $\sigma _{a}$ are the weight coefficients that control the influence of $\mathbf{n}_{r}$ and $\mathbf{n}_{a}$, respectively.

The perturbed parallel single-cell data $\hat{\textbf{X}}^{r}$ and $\hat{\textbf{X}}^{a}$ is fed into a multi-modal autoencoder, wherein it is embedded into a lower-dimensional feature space, as represented by the following equation: 


(2) \begin{align*}& \begin{aligned} \mathbf{Z}^{r} ={f}_{e}^{r}(\hat{\textbf{X}}^{r});\mathbf{Z}^{a} ={f}_{e}^{a}(\hat{\textbf{X}}^{a}), \end{aligned}\end{align*}

here ${f}_{e}^{r}(\cdot )$ and ${f}_{e}^{a}(\cdot )$ correspond to the encoder mappings for scRNA and scATAC data, respectively. While $\mathbf{Z}^{r}$ and $\mathbf{Z}^{a}$ represent the resulting low-dimensional embeddings from both modalities. These embeddings are subsequently transformed by the SAN module, resulting in the generation of the final shared embedding, denoted as $\tilde{\textbf{Z}}$.

Upon this basis, we have introduced the ZINB distribution to estimate the distribution of single-cell data [25–27]. Despite the ZINB loss not being specifically designed for scATAC-seq data, its proficiency in addressing over-dispersion and data sparsity renders it an appropriate selection. Following the approach of Lin *et al*., this work models both scRNA and scATAC data using ZINB loss [24]. Nevertheless, this study does not claim that ZINB distribution is the optimal distribution for scATAC data, researchers are encouraged to consider a loss function that may be more appropriate for scATAC data. Prior to constructing the ZINB distribution, the Negative Binomial (NB) distribution is initially computed, which is a type of discrete distribution. Since this work involves two modalities of data, we will elucidate the equation using an example from the scRNA modality $\mathbf{X}^{r}$: 


(3) \begin{align*} & \textrm{NB}(\mathbf{X}^{r}\mid\mu,\theta)=\frac{\Gamma(\mathbf{X}^{r}+\theta)}{\mathbf{X}^{r} ! \Gamma(\theta)}\left(\frac{\theta}{\theta+\mu}\right)^{\theta}\left(\frac{\mu}{\theta+\mu}\right)^{\mathbf{X}^{r}}, \end{align*}

(4) \begin{align*}& \operatorname{ZINB}(\mathbf{X}^{r}\mid \pi, \mu, \theta)=\pi \delta_{0}(\mathbf{X}^{r})+(1-\pi) \operatorname{NB}(\mathbf{X}^{r}),\end{align*}

here $\pi $, $\mu $, $\theta $ denote the dropout rate, dispersion degree, and mean, respectively. Deviating from a conventional autoencoder, the ZINB-based denoising autoencoder incorporates three separate fully connected layers that are connected to the last layer of the decoding network. This architecture aims to estimate the parameters ${\pi ,\mu ,\theta }$ within the shared embedding $\tilde{\textbf{Z}}$, which is denoted as below: 


(5) \begin{align*}& \begin{array}{c} {\Pi = {\mathop{\textrm{sigmoid}}\nolimits} \left( {{\textbf{W}}_{\pi}^{r}f_{d}^{r}(\widetilde{\textbf{Z}})} \right);}\\{M = \exp \left( {{\textbf{W}}_{\mu}^{r}f_{d}^{r}(\widetilde{\textbf{Z}})} \right);}\\{\Theta = \exp \left( {{\textbf{W}}_{\theta}^{r}f_{d}^{r}(\widetilde{\textbf{Z}})} \right),} \end{array}\end{align*}

${f}_{d}^{r}$ represents a fully connected decoding neural network. $\mathbf{W}_{\pi }^{r}$, $\mathbf{W}_{\mu }^{r}$ and $\mathbf{W}_{\theta }^{r}$ are three learnable weight matrices corresponding to three parameters in the ZINB distribution. $\Pi , M, \Theta $ are parameter matrices representing the dropout rate, mean and dispersion, respectively. It is worth noting that the dropout rate typically ranges between 0 and 1, which accounts for why we employ the sigmoid function $\operatorname{sigmoid}(\cdot )$ for $\Pi $. Similarly, we apply the exponential function $\exp (\cdot )$ to the other two parameters because of their non-negativity. Finally, the negative log-likelihood of the ZINB distribution is defined as the reconstruction loss for the input data $\mathbf{X}^{r}$, and its mathematical form is as follows: 


(6) \begin{align*}& \begin{aligned} \mathcal{L}_{z}^{r} = -\log(\operatorname{ZINB} (\mathbf{X}^{r}\mid \pi, \mu, \theta)), \end{aligned}\end{align*}

the computational process for scATAC is similar to that of scRNA and can be represented as follows: 


(7) \begin{align*}& \begin{aligned} \mathcal{L}_{z}^{a} = -\log(\operatorname{ZINB} (\mathbf{X}^{a}\mid \pi, \mu, \theta)), \end{aligned}\end{align*}

ultimately, for parallel scRNA and scATAC data, the overall reconstruction loss of ZINB-based denoising autoencoder is defined as follows: 


(8) \begin{align*}& \begin{aligned} \mathcal{L}_{rec} = \mathcal{L}_{z}^{r} + \mathcal{L}_{z}^{a}. \end{aligned}\end{align*}

### Skip aggregation network

Given the disparity in data richness between the scRNA and scATAC modalities, parallel analysis necessitates an effective aggregation method to handle the multi-modal data. Therefore, we introduce the SAN module that begins with the concatenation of the embeddings from both modalities, as depicted below: 


(9) \begin{align*}& \begin{aligned} \mathbf{Z} =[\mathbf{Z}^{r}, \mathbf{Z}^{a}]. \end{aligned}\end{align*}

Inspired by the transformer architecture [28–30], we have designed a similar structure to map the concatenated embeddings $\mathbf{Z}$ into three separate feature spaces. The mapping process is as follows: 


(10) \begin{align*}& \begin{aligned} \mathbf{H}_{1} =\mathbf{Z}\mathbf{W}_{1}^{t}; \mathbf{H}_{2} =\mathbf{Z}\mathbf{W}_{2}^{t}; \mathbf{H}_{3} =\mathbf{Z}\mathbf{W}_{3}^{t}, \end{aligned}\end{align*}

here, $\mathbf{W}_{1}^{t}$, $\mathbf{W}_{2}^{t}$ and $\mathbf{W}_{3}^{t}$ represent three weight matrices used for the mapping transformation, while $\mathbf{H}_{1}$, $\mathbf{H}_{2}$ and $\mathbf{H}_{3}$ denote three obtained embeddings via the mapping process. Subsequently, $\mathbf{H}_{1}$ and $\mathbf{H}_{2}$ are utilized to compute a global relationship matrix $\mathbf{H}_{s}$: 


(11) \begin{align*}& \begin{aligned} \mathbf{H}_{s}=\operatorname{softmax}\left(\frac{\mathbf{H}_{1}\mathbf{H}_{2}^{T}}{\sqrt{d}}\right), \end{aligned}\end{align*}

$d$ represents the dimension of the embedding $\mathbf{Z}$. Afterward, the preserved embedding $\mathbf{H}_{3}$ is enhanced by global relationship matrix $\mathbf{H}_{s}$, simultaneously combined with $\mathbf{Z}$ through skip connections to prevent network degradation. The mathematical process is as follows: 


(12) \begin{align*}& \begin{array}{l} \hat{\textbf{Z}} = {\mathbf{W}^{h}}\left( \textbf{Z}+\mathbf{H}_{s}\mathbf{H}_{3}\right) + {\mathbf{b}}, \end{array}\end{align*}

where $\mathbf{W}^{h}$ denotes weight matrix for the skip transformation, $\mathbf{b}$ represents the corresponding bias. Then we obtained the aggregated embedding $\hat{\textbf{Z}}$. To preserve the rich information of scRNA and prevent degradation of the aggregated representation, we concatenate the aggregated representation $\hat{\textbf{Z}}$ with the original scRNA embedding $\mathbf{Z}^{r}$, this forms the basis of the proposed skip module. Such an adjustment effectively transforms the aggregation module into a fine-tuning mechanism tailored for scRNA data. Consequently, this approach not only utilizes information from multiple modalities but also ensures that the final cellular representation is robust against the sparse data characteristic of a single modality. The formula is as follows: 


(13) \begin{align*}& \begin{aligned} \tilde{\textbf{Z}} =[\mathbf{Z}^{r}, \hat{\textbf{Z}}]. \end{aligned}\end{align*}

### Joint optimizing module

During the training process, reconstruction and clustering loss are employed for joint optimization. We minimize the following overall objective function: 


(14) \begin{align*}& \begin{aligned} \mathcal{L}_{f} = \lambda_{1}\mathcal{L}_{rec} + \lambda_{2}\mathcal{L}_{clu} \end{aligned}\end{align*}

where $\mathcal{L}_{rec}$ and $\mathcal{L}_{clu}$ represent the clustering loss and reconstruction loss, respectively, while $\lambda _{1}$ and $\lambda _{2}$ are two hyperparameters that balance their contributions. The reconstruction loss $\mathcal{L}_{rec}$ has been previously described in detail. On the other hand, the clustering loss, $\mathcal{L}_{clu}$, can be further decomposed into two components: the Kullback–Leibler (KL) divergence loss and the deep $k$-means loss.

#### KL divergence on the cell representations

During the clustering process, cells with similar features are assigned to the same cluster. In this work, we employ the KL divergence loss to further enhance the correlation among similar cells. Following the previous approach [31–33], we utilize the Student’s t-distribution to depict the pairwise similarity between cell i and cell j, as presented below: 


(15) \begin{align*}& q_{i j}=\frac{\left(1+\left\|\tilde{\textbf{Z}}_{i}-\tilde{\textbf{Z}}_{j}\right\|^{2}\right)^{-1}}{\sum_{l\neq i}\left(1+\left\|\tilde{\textbf{Z}}_{i}-\tilde{\textbf{Z}}_{l}\right\|^{2}\right)^{-1}},\end{align*}

here, $\tilde{\textbf{Z}}_{i}$ and $\tilde{\textbf{Z}}_{j}$ denote the emdedding of cell $i$ and $j$, $q_{ij}$ represents the soft assignment, measuring the pairwise similarity between two cells, i and j. Additionally, $p_{ij}$ is the target distribution, constructed based on $q_{ij}$. This construction is designed to enhance or diminish the affinities between cells with higher and lower similarities, respectively. The computational process is as follows: 


(16) \begin{align*}& \begin{aligned} p_{i j}=\frac{q_{i j}^{2} / \sum_{i=1}^{n} q_{i j}}{\sum_{l\neq i}\left(q_{i l}^{2} / \sum_{l\neq i} q_{i l}\right)}. \end{aligned}\end{align*}

Upon acquiring two distributions, we formulate the KL divergence loss as a means to converge $\mathbf{Q}$ towards $\mathbf{P}$. The expression for the KL loss function is as follows: 


(17) \begin{align*}& \begin{aligned} \mathcal{L}_{kl}=\operatorname{KL}(\mathbf{P}||\mathbf{Q})=\sum_{i}\sum_{j} p_{i j}\log \frac{p_{i j}}{q_{i j}}. \end{aligned}\end{align*}

#### Deep *k*-means clustering

In the bottleneck layer, also known as the hidden layer, we performed unsupervised clustering and the clustering loss is defined as follows: 


(18) \begin{align*}& \begin{aligned} \mathcal{L}_{dk}=\sum_{i=1}^{N}\sum_{j=1}^{K} w_{i j} f(\tilde{\textbf{Z}}_{i},\mathbf{V}_{j}), \end{aligned}\end{align*}

where, $\mathbf{V}_{j}$ represents the $j$-th cluster center, $f(\cdot )$ calculates the Euclidean distance between the cell and the cluster center. While $w_{i j}$ represents the weight of distance. To ensure gradient smoothness, the Gaussian kernel is employed for the transformation of feature projections, following the procedure outlined below: 


(19) \begin{align*}& \begin{aligned} \tilde{w}_{i j}=\frac{\exp(-f(\tilde{\textbf{Z}}_{i},\mathbf{V}_{j}))}{\sum_{k=1}^{K}\exp(-f(\tilde{\textbf{Z}}_{i},\mathbf{V}_{k}))}. \end{aligned}\end{align*}

To facilitate convergence, an additional inflation operation is incorporated for the weight $\tilde{w}_{i j}$: 


(20) \begin{align*}& \begin{aligned} {w}_{i j}=\frac{\tilde{w}_{i j}^{2}}{\sum_{k=1}^{K}\tilde{w}_{i j}^{2}}. \end{aligned}\end{align*}

Then, we amalgamated the KL divergence loss and the deep $k$-means distance loss to form the ultimate clustering loss $\mathcal{L}_{clu}$: 


(21) \begin{align*}& \begin{aligned} \mathcal{L}_{clu} = \mathcal{L}_{kl} + \mathcal{L}_{dk}. \end{aligned}\end{align*}

### Datasets

In this parallel clustering analysis, we conducted comprehensive experiments on five authentic multi-modal single-cell datasets: BMNC (https://www.ncbi.nlm.nih.gov/geo), PBMC (https://www.10xgenomics.com/resources/datasets), SLN111 (https://github.com/YosefLab/totalVI_reproducibility), SMAGE-10K^2^, SMAGE-3K^2^. The data sources have been delineated in the footnotes, and corresponding cell type labels were downloaded. The cluster number was determined by the categories of the downloaded cell type labels. All of these datasets encompass both scRNA and scATAC sequencing for the same batch of cells. In cases where the datasets have already undergone dimensionality reduction by the original authors, we will employ their processed forms. For datasets that have not yet been dimensionally reduced, we achieve standardization by limiting the feature count to 2000, thus ensuring consistency. An overview of the dataset information, including the number of cells, dimensions of the scRNA data, dimensions of the scATAC data and the number of clusters, is provided in Table 2.

**Table 2:** The summary of datasets

Dataset	Samples	scRNA.dim	scATAC.dim	Clusters
BMNC	30 672	1000	25	27
PBMC	3762	1000	49	16
SLN111	16 828	1000	112	35
SMAGE-10K	11 020	2000	2000	12
SMAGE-3K	2585	2000	2000	14

### Evaluation metrics

This work employed two widely used evaluation metrics, adjusted Rand index (ARI) and normalized mutual information (NMI), to evaluate the clustering performance.

ARI is a measure of similarity between two clusterings, which ranges between −1 and 1, with closer to 1 indicating higher consistency. It can be formulated as follows: 


(22) \begin{align*}& \begin{aligned} \textrm{ARI} = \frac{\sum_{ij}\binom{n_{ij}}{2}-[\sum_{i}\binom{a_{i}}{2}\sum_{j}\binom{b_{j}}{2}]/\binom{n}{2}}{\frac{1}{2}[\sum_{i}\binom{a_{i}}{2}+\sum_{j}\binom{b_{j}}{2}]-[\sum_{i}\binom{a_{i}}{2}\sum_{j}\binom{b_{j}}{2}]/\binom{n}{2}}. \end{aligned}\end{align*}

Furthermore, NMI is a normalized mutual information metric used to measure the shared information between two clusterings, which ranges between 0 and 1, with closer to 1 indicating higher similarity. Its mathematical representation is as follows: 


(23) \begin{align*}& N M I=\frac{2 M I(U, V)}{H(U)+H(V)}.\end{align*}

### Implementation details

We employ PyTorch (version 1.13.1) in Python 3.7 to implement scEMC. The encoding layers of the ZINB-based denoising autoencoder are set (256, 64, 32, 8), with a bottleneck layer size of 8 for both scRNA and scATAC. The aggregated bottleneck layer size is 24, while the batch size is set to 256. Initially, we conduct the pretraining for 400 epochs, followed by 5000 epochs of training. These experiments are performed on a personal computer running the Linux operating system, which is configured with an i9-12900KF CPU, 64 GB of RAM and a GeForce RTX 3070Ti GPU. It is important to note that our algorithm utilizes $k$-means, so the user needs to manually specify the number of clusters before running the algorithm. For the baseline methods, we conducted the $k$-means and spectral clustering algorithms utilizing the scikit-learn package. Regarding the other comparative methods, we followed the implementations as delineated in their respective official repositories. For all methods employed, parameter settings were maintained as per the default configurations.

## RESULTS

### scEMC attains outstanding clustering performance

To comprehensively evaluate the clustering performance of our scEMC, in this work, we conduct thorough experimentation across five multi-modal single-cell datasets, along with the inclusion of eight competitive methods.

These competitive methodologies can be categorized into three groups, multi-modal clustering methods: scMVAE, scMCs, DCCA; single-modal clustering methods: scDSC, scDeepCluster, DESC; foundational clustering methods: spectral clustering and $k$-means clustering. A brief introduction to these approaches is presented below:

- **scMVAE** [17]: deep-joint-learning analysis model of single-cell transcriptome and open chromatin accessibility data.
- **scMCs** [22]: scMCs: a framework for single-cell multi-omics data integration and multiple clusterings.
- **DCCA** [21]: deep cross-omics cycle attention model for joint analysis of single-cell multi-omics data.
- **scDSC** [12]: deep structural clustering for scRNA data jointly through autoencoder and graph neural network.
- **scDeepCluster** [10]: clustering scRNA data with a model-based deep learning approach.
- **DESC** [9]: deep learning enables accurate clustering with batch effect removal in scRNA analysis.
- **Spectral clustering** [7]: a tutorial on spectral clustering.
- **
$k$ -means** [6]: Algorithm AS 136: a $k$-means clustering algorithm.

As depicted in Table 3, the clustering performance of scEMC and the eight competitive methods is quantitatively evaluated by ARI and NMI. The results indicate that scEMC surpasses other clustering algorithms significantly. Over a series of 10 evaluations, it consistently achieved the top position 9 times, yielding only the second position in NMI for the PBMC dataset. It remains a fact that no algorithm can attain perfection in every scenario. Nevertheless, scEMC consistently showcases exceptional clustering performance in the majority of situations.

**Table 3:** Clustering result comparison for five datasets.

Datasets	BMNC		PBMC		SLN111		SMAGE-10K		SMAGE-3K	
Metrics	ARI	NMI	ARI	NMI	ARI	NMI	ARI	NMI	ARI	NMI
$k$ -means	0.5205	0.7443	0.4768	0.6734	0.2629	0.5659	0.465	0.5861	0.5109	0.5807
Spectral	0.4497	0.6919	0.5018	0.7022	0.4315	0.6060	0.4982	0.5679	0.5389	0.5989
DESC	0.5125	0.6872	0.5125	0.6872	0.4607	0.5712	0.3263	0.5322	0.5360	0.5664
scDeepCluster	0.5676	0.7572	0.5676	0.7572	0.3482	0.5591	0.3518	0.5604	0.3929	0.5740
scDSC	0.6193	0.6504	0.6193	0.6504	0.2992	0.4308	0.5102	0.5314	0.5514	0.6189
DCCA	0.4912	0.7277	0.4912	0.7277	0.2611	0.5809	0.3866	0.5511	0.2984	0.5473
scMCs	0.1841	0.3906	0.1841	0.3906	0.0947	0.3088	0.2471	0.3598	0.2505	0.4255
scMVAE	0.4225	0.7060	0.5437	0.6983	0.2161	0.5936	0.3430	0.5726	0.3616	0.5794
scEMC(ours)	0.6480	0.7603	0.6289	0.7325	0.4654	0.6149	0.6953	0.6636	0.6419	0.6572

### scEMC effectively integratessparse information from the scATAC modality

The motivation for our study arises from the observation that the application of data from multiple modalities in multi-modal clustering analysis does not consistently yield improved results. A deeper investigation into this pattern revealed that the quality of scRNA modality data generally exceeds that of scATAC data. As a result, the comparatively lower quality of scATAC data can negatively influence the overall performance of the model. To solve this issue, we developed the SAN network, which shifts our model from an equal integration strategy to a refined tuning mechanism primarily based on scRNA modality data.

**Figure 2:**
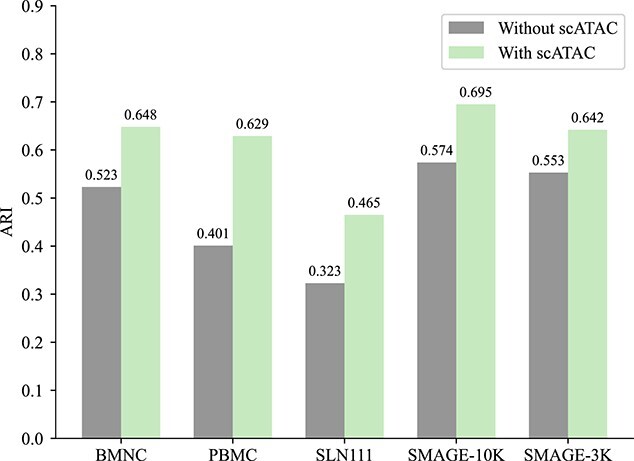
Assess the model’s performance both with and without scATAC information using the ARI.

**Figure 3:**
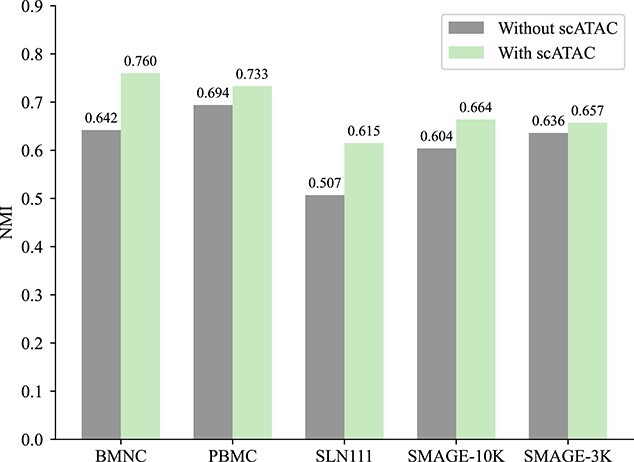
Assess the model’s performance both with and without scATAC information using the NMI.

To assess the SAN network’s effectiveness in leveraging the lower-quality scATAC data, we executed comparative experiments on the five datasets involved in our study. These experiments were divided into two categories: one without incorporating scATAC modality information and the other including all data. The outcomes, illustrated in Figures 2 and 3, reveal a noticeable decrease in model performance when scATAC modality information is excluded. This finding highlights that the SAN network not only diminishes the adverse effects of low-quality scATAC data on the model but also well consolidates sparse information from the scATAC modality, thereby improving clustering accuracy.

### scEMC learns effective cell representations in latent space

In this research, plenty of computations take place within the latent space. Consequently, the quality of cell representations directly exerts influence on clustering performance.

To investigate whether scEMC has learned high-quality cell representations, we retained the hidden layers of scEMC and its various variants, visualizing them through t-SNE on the PBMC dataset. These variants included four different absences: removal of the aggregation module, skip connections with scRNA, clustering loss, and reconstruction loss. For the sake of illustration, we use the abbreviation w.o. to signify the absence of these modules.

**Figure 4:**
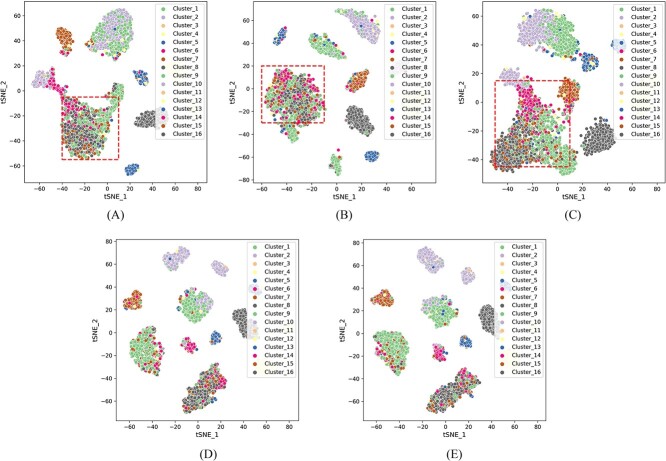
2D t-SNE visualization showcasing the clustering quality disparities among embeddings in the absence of various modules, on the PBMC dataset. (**A**) w.o. aggregation module. (**B**) w.o. skip module. (**C**) w.o. clustering loss. (**D**) w.o. reconstruction loss. (**E**) scEMC with all modules preserved.

As shown in Figure 4, we observed that once the skip connection with scRNA data was removed, the quality of the learned cell representations drastically declined, resulting in chaotic clusters that make it challenging to distinguish between different cell types. Simultaneously, when the structural aggregation module is removed, cells that do not belong to the same class are grouped into one cluster. This implies that the structural aggregation module effectively enhances the quality of the learned embeddings. Furthermore, the removal of clustering loss or reconstruction loss leads to a certain degree of degradation in cell representations’ quality, demonstrating the effectiveness of the loss functions we have employed in optimizing the cell representations.

### Ablation study

To further investigate the individual impacts of proposed modules on the overall performance, we conducted comprehensive ablation experiments.

Specifically, we constructed two sets of variants of scEMC and compared their clustering performance. The first set of variants was created to validate the effectiveness of the network structure. In this set, we devised two variants: one that removed the structural aggregation mechanism and another that eliminated skip connections with the scRNA data. The results are presented in Table 4, and from the results, it is evident that both the removal of the structural aggregation mechanism and skip connections with the scRNA data resulted in a significant degradation in performance. This indicates that the structural aggregation mechanism effectively integrates information from both modalities, while skip connection with the scRNA data effectively prevents network degradation.

**Table 4:** Ablation study of skip and aggregation module.

Datasets	Metric	w.o.Skip	w.o.Aggregation	scEMC
BMNC	ARI	0.5207	0.5496	0.6480
	NMI	0.7309	0.7166	0.7603
PBMC	ARI	0.4776	0.4644	0.6289
	NMI	0.7187	0.6982	0.7325
SLN111	ARI	0.4326	0.3780	0.4654
	NMI	0.6033	0.5487	0.6149
SMAGE-10K	ARI	0.5400	0.5618	0.6953
	NMI	0.6184	0.6363	0.6636
SMAGE-3K	ARI	0.5657	0.5883	0.6419
	NMI	0.6259	0.6285	0.6572

The second set of variants aimed to explore the effectiveness of the optimization modules. In this set of variants, we separately removed the reconstruction loss and the clustering loss. As shown in Table 5, scEMC exhibited the best performance, while the other two variants demonstrated a noticeable decline in performance. This highlights the critical importance of both clustering and reconstruction losses in the optimization process, indicating that the constraint losses we have introduced effectively optimize the cell representations.

**Table 5:** Ablation study of optimizing module.

Datasets	Metric	w.o.$\mathcal{L}_{r}$	w.o.$\mathcal{L}_{c}$	scEMC
BMNC	ARI	0.6201	0.4054	0.6480
	NMI	0.7414	0.5846	0.7603
PBMC	ARI	0.6145	0.4058	0.6289
	NMI	0.7277	0.5977	0.7325
SLN111	ARI	0.4592	0.2768	0.4654
	NMI	0.6144	0.5398	0.6149
SMAGE-10K	ARI	0.6211	0.5021	0.6953
	NMI	0.6222	0.5917	0.6636
SMAGE-3K	ARI	0.5875	0.3974	0.6419
	NMI	0.6362	0.5751	0.6572

### Convergence analysis

**Figure 5:**
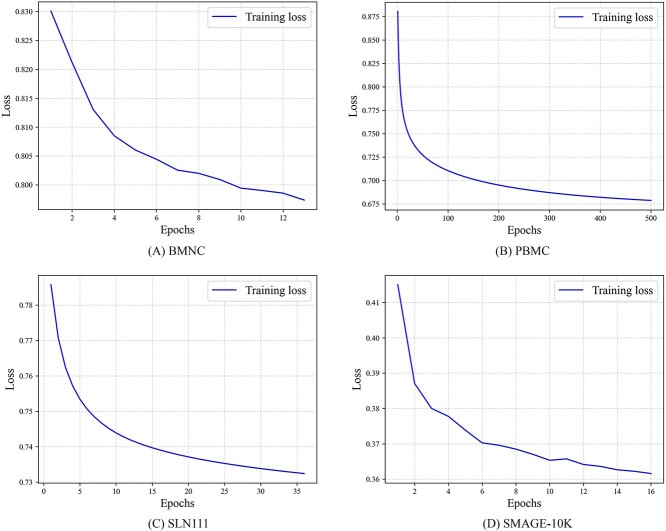
The descent process of the loss function on four benchmark datasets. (**A**) BMNC, (**B**)PBMC, (**C**) SLN111, (**D**) SMAGE-10K.

To intuitively assess whether the model has been effectively optimized and achieved convergence, we saved the loss values at each epoch and plotted the descent curves. As depicted in Figure 5, it is evident that the loss values on all four datasets exhibit monotonic decreasing trends until convergence, indicating that the model has been adequately trained.

The descent curves do not further extend into horizontal lines, which may be attributed to the early stopping mechanism we incorporated into the algorithm for the sake of computational efficiency. Once the model converges to the threshold, the training process is prematurely terminated. Nevertheless, the results in Figure 5 robustly confirm the effectiveness of optimization and the convergence of our algorithm.

### Parameter analysis

In the previous section, we built two hyperparameters, denoted as $\lambda _{1}$ and $\lambda _{2}$, to measure the contribution between clustering loss and reconstruction loss.

**Figure 6:**
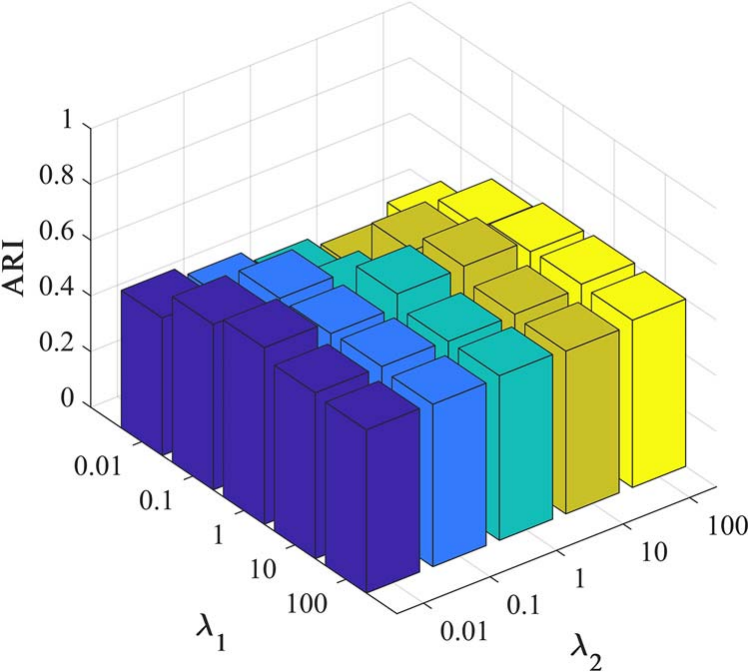
Investigation of hyperparameter $\lambda _{1}$ and $\lambda _{2}$ by ARI.

**Figure 7:**
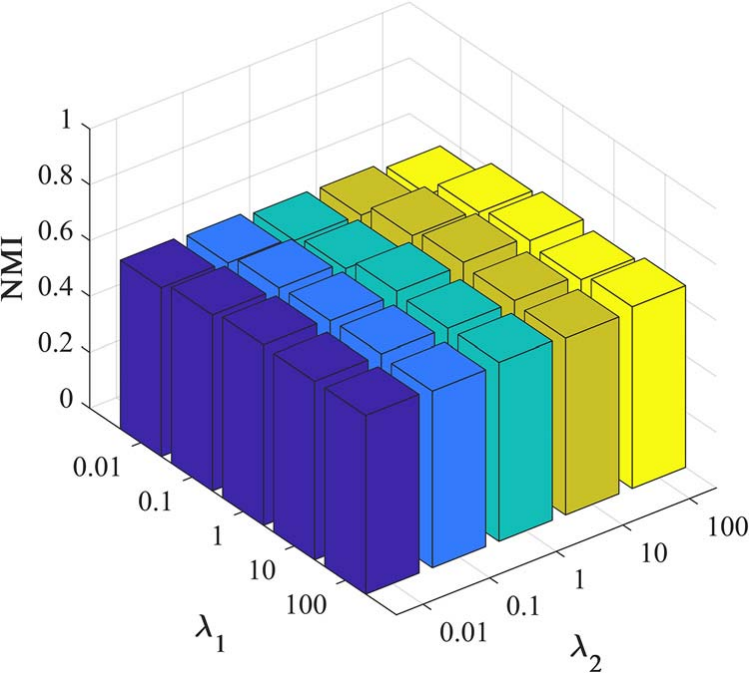
Investigation of hyperparameter $\lambda _{1}$ and $\lambda _{2}$ by NMI.

Here, we comprehensively assessed the influence of these hyperparameters on the clustering performance of scEMC. The experiments were conducted under various parameter sets, with both parameters ranging from (0.01, 0.1, 1, 10, 100). The three-dimensional visualizations of the results are presented in Figures 6 and 7.

From Figures 6 and 7, it becomes apparent that the NMI performance of the proposed scEMC algorithm remains not sensitive to the parameter values within this range. The performance exhibits minimal fluctuations, with only a marginal decline observed when $\lambda _{1}$ is set to 0.01 and $\lambda _{2}$ is set to 100. Conversely, Figures 6 and 7 reveals that the ARI performance of the scEMC algorithm is sensitive to $\lambda _{1}$ within the range of 0.01 to 1. Optimal performance is achieved at $\lambda _{1}$=1 and $\lambda _{2}$=10. This phenomenon might be attributed to the fact that ARI is based on the consistency in categorizing paired elements, whereas NMI relies on information sharing, thereby rendering it relatively insensitive to changes in parameters when compared to ARI. Based on experience, we configure $\lambda _{1}$ and $\lambda _{2}$ in accordance with this optimal parameter setting.

## CONCLUSION

In conclusion, we have developed an effective parallel clustering method, scEMC, tailored for scRNA and scATAC data. It leverages the transformer architecture to learn cross-modal global structural information from parallel single-cell data and facilitates the fusion of cross-modal information. Additionally, by incorporating skip connections that link with scRNA modality data, scEMC prevents the network from degrading. This skip mechanism effectively preserves richer scRNA data, while the designed denoising autoencoder based on ZINB optimally fits single-cell data and refines the cell representations. Experimental results demonstrate that our model outperforms other methods in terms of clustering performance.

Furthermore, there remain certain limitations that necessitate our attention. Currently, our proposed framework primarily considers the parallel analysis of scRNA and scATAC data. More sequencing modalities can be integrated into our framework in the future. Additional fusion strategies, such as concatenation and ensemble learning [34–36], can be incorporated to enhance the aggregation capabilities of our framework. Additionally, the design of a more discriminative network structure might be a potentially effective direction to improve this model.

**Key Points**

- We propose an effective parallel clustering framework scEMC, which mitigates the impact of unbalanced information richness of scRNA and scATAC data.
- Different from previous methods, we have introduced a pioneering SAN module that incorporates transformer structure to learn the global structural relationships between diverse feature spaces, facilitating aggregation across different modalities. Moreover, we create a skip connection between the aggregated representation and the scRNA modality data to safeguard the network from degradation.
- By leveraging a denoising autoencoder based on the ZINB loss, scEMC enables the network to fit the real distribution of single-cell data. Extensive experiments demonstrate the excellence of scEMC, surpassing the other benchmark methods.

## Data Availability

The datasets and code can be publicly accessed in Repository https://github.com/DayuHuu/scEMC.

## Author Biographies

**Dayu Hu** is currently pursuing a PhD degree at the National University of Defense Technology (NUDT). Before joining NUDT, he got his BSc degree at Northeastern University (NEU). His current research interests include graph learning and bioinformatics. He has published several papers and served as PC member/Reviewer in highly regarded journals and conferences such as ACM MM, AAAI, TNNLS, TKDE, TCBB, etc.

**Ke Liang** is currently pursuing a PhD degree at the National University of Defense Technology (NUDT). Before joining NUDT, he got his BSc degree at Beihang University (BUAA) and received his MSc degree from the Pennsylvania State University (PSU). His current research interests include knowledge graphs, graph learning, and healthcare AI. He has published several papers in highly regarded journals and conferences such as SIGIR, AAAI, ICML, ACM MM, IEEE TNNLS, IEEE TKDE, etc.

**Zhibin Dong** is currently pursuing the PhD degree with the National University of Defense Technology (NUDT), Changsha, China. He has published several papers and served as a Program Committee (PC) member or a reviewer for top conferences, such as IEEE Conference on Computer Vision and Pattern Recognition (CVPR), IEEE International Conference on Computer Vision (ICCV), Association for Computing Machinery's Multimedia Conference (ACM MM), Association for the Advancement of Artificial Intelligence Conference (AAAI), and the IEEE Transactions on Neural Networks and Learning Systems (TNNLS). His current research interests include graph representation learning, deep unsupervised learning, and multi-view clustering.

**Jun Wang** received the MS degree from China University of Geosciences, Wuhan, China, in 2023. Currently, he is pursuing his PhD degree at School of Computer Science, National University of Defense Technology. His research interest is multiview learning.

**Yawei Zhao** is now working at Medical Big Data Research Center, Chinese PLA General Hospital, Beijing, 100853, China. He received the PhD degree in Computer Science from the National University of Defense Technology, China in 2020. His research interests include time-series analysis, medical data analysis, and federated learning.

**Kunlun He** received his MD degree from The 3rd Military Medical University, Chongqing, China in 1988, and PhD degree in Cardiology from Chinese PLA Medical school, Beijing, China in 1999. He worked as a postdoctoral research fellow at the Division of circulatory physiology of Columbia University from 1999 to 2003. He is the director and professor of the Medical Big Data Research Center, Chinese PLA General Hospital, Beijing, China. His research interests include big data and artificial intelligence of cardiovascular disease.
